# Effectiveness of a Person-Centered and Culturally Sensitive Course of Treatment in Arabic-, Turkish-, and Urdu-Speaking Individuals With Type 2 Diabetes (the ACCT2 Study): Protocol for a Pragmatic Randomized Controlled Trial

**DOI:** 10.2196/67319

**Published:** 2025-06-05

**Authors:** Natasja Bjerre, Lene Christensen, Christina Hoeiberg, Cecilie Ottosson, Mie Klarskov Jensen, Nanna Kildsig, Trine Kjeldgaard Møller, Anne-Ditte Termannsen, Bettina Ewers, Britt Hollender-Schou, Rikke Molin Grue, Ulla Bjerre-Christensen, Solveig Jansen, Kamran Akram

**Affiliations:** 1 Department of Education Steno Diabetes Center Copenhagen Copenhagen University Hospital Herlev Denmark; 2 Centre for Diabetes Municipality of Copenhagen Copenhagen Denmark; 3 Department of Diabetes Treatment Steno Diabetes Center Copenhagen Copenhagen University Hospital Herlev Denmark

**Keywords:** immigrants, migrants, diabetes management, ethnic minorities, trial, well-being, public health, type 2 diabetes, culturally-sensitive, randomized controlled trial

## Abstract

**Background:**

Individuals with non-Western backgrounds consistently exhibit a higher risk of type 2 diabetes (T2D) than ethnic Danes. Factors such as health behaviors, limited health care access, and social determinants of health often contribute to this disparity. Culturally sensitive interventions are crucial; however, effective interventions for managing T2D in non-Western populations remain limited.

**Objective:**

This study aims to examine the effect of a 1-year person-centered and culturally sensitive intervention on improving glycemic control (hemoglobin A_1c_ [HbA_1c_]) in Arabic-, Turkish-, or Urdu-speaking individuals with T2D. The secondary objectives are to improve diabetes management and overall well-being.

**Methods:**

This study is designed as a 2-arm randomized controlled trial. A total of 96 women and men with T2D (HbA_1c_≥53 mmol/mol) speaking either Arabic, Turkish, or Urdu as their native language will be randomized for 1 year to a health care professional–assisted intervention group (person-centered and culturally sensitive course of treatment) or a control group (usual care) in a 1:1 ratio in Denmark. Assessments are scheduled at baseline and 1 year. The primary outcome is HbA_1c_, while lipids, blood pressure, and patient-reported outcomes, including well-being, diabetes management, health literacy, and use of and adherence to diabetes medication, are secondary outcomes. Feasibility and satisfaction are evaluated through interviews. The study is approved by the Ethics Committee of the Capital Region of Denmark (H-23042245).

**Results:**

A 5.0-mmol/mol change in HbA_1c_ is the minimally important difference, requiring 88 participants. To allow for uncertainties and dropouts, the total was increased to 96. As of January 2025, a total of 88 participants have been recruited. Recruitment was completed in March 2025. Data collection will continue until December 2025, with the first results expected by March 2026.

**Conclusions:**

This study will contribute to the limited knowledge regarding the effects of person-centered and culturally sensitive treatment approaches for T2D in Arabic-, Turkish-, or Urdu-speaking individuals. The study uses a robust methodological design and will present an alternative avenue for managing T2D and improving overall well-being. The study offers valuable insights into the experiences of participants and health care professionals, including potential obstacles and strategies for implementation in outpatient clinics.

**Trial Registration:**

ClinicalTrials.gov NCT06147245; https://clinicaltrials.gov/study/NCT06147245

**International Registered Report Identifier (IRRID):**

DERR1-10.2196/67319

## Introduction

European studies have steadily indicated that immigrant groups born in non-Western countries are at a significantly higher risk of developing type 2 diabetes (T2D) than the majority population [1-3]. In Denmark, immigrants from the Middle East exhibit a T2D incidence rate 2.5 times higher than native Danes [4,5] and face increased risks of complications [2,3,6]. Individuals with non-Danish ethnic backgrounds have poorer glycemic control [7,8], and a lower proportion receive diabetes monitoring and care within recommended intervals outlined in the Danish Diabetes guidelines [7].

Individuals with non-Western backgrounds experience difficulties in managing their diabetes effectively. This is due to factors such as health behavior, access to health care, and social determinants, as well as low health literacy, cultural norms, and language barriers that hinder access to and comprehension of information and treatment of diabetes [4,9-13]. Other factors include misconceptions about diabetes, inadequate adherence to medication regimens, lifestyle factors, limited social support, and lack of cultural competencies among health care professionals (HCPs) [1,13-16]. This emphasizes a need for culturally appropriate interventions to improve access to health care and support, but existing interventions often overlook these cultural considerations [17,18].

Resnicow et al [17] define cultural sensitivity as incorporating cultural characteristics, experiences, norms, values, behavioral patterns, beliefs, and environmental and social forces of a target population to design, deliver, and evaluate interventions. Despite some interventions tailored for cultural minority groups that have shown positive effects on hemoglobin A_1c_ (HbA_1c_) [19,20], recent research highlights a significant gap in person-centered and culturally sensitive interventions for individuals with T2D. Despite guidelines recommending a person-centered approach, there is a disconnect between theory and practice [21], and current diabetes self-management education and support programs often lack cultural competence and literacy-sensitive recommendations [22-24]. These findings underscore the need for more research on culturally sensitive, person-centered interventions to improve diabetes management and reduce health disparities, particularly in outpatient clinics [25-27]. This study aims to explore the effectiveness of a person-centered, culturally sensitive intervention for individuals with T2D speaking Urdu, Arabic, or Turkish to improve glycemic control, as well as diabetes management, health literacy, and overall well-being by using a pragmatic randomized controlled trial (RCT) design [28,29].

## Methods

### Objectives

The overall objective of this study is to examine the effect of a 1-year, person-centered and culturally sensitive intervention on alterations in glycemic control (HbA_1c_) in Arabic-, Turkish-, or Urdu-speaking individuals. Secondary objectives describe changes in cardiometabolic risk factors and patient-reported outcomes, including well-being, diabetes management, health literacy, and adherence to diabetes medication.

### Hypotheses

We hypothesize that a 1-year, person-centered and culturally sensitive intervention added to standard care will be superior for reducing HbA_1c_ in Arabic-, Turkish-, or Urdu-speaking individuals with T2D. Superiority will be claimed if the baseline-corrected difference between the 2 groups is equal to or surpasses the minimal important difference (5 mmol/mol) in favor of the intervention group and if the *P*<.05.

### Participants

Women and men aged 75 years or younger with T2D whose native language is Urdu, Arabic, or Turkish will be considered eligible based on the specified inclusion and exclusion criteria.

### Eligibility Criteria

The inclusion and exclusion criteria are listed in Textbox 1. The eligibility criteria, including the age limit, were carefully selected based on several considerations. The language criteria were chosen to focus on the largest language groups at Steno Diabetes Center Copenhagen (SDCC), as individuals who speak fluent Danish generally face fewer challenges in managing their treatment and T2D compared with those with limited Danish proficiency. Regarding the age limit, participants older than 75 years were excluded as they are typically offered a different, combined approach to care. However, we recognize that older individuals need support, and it is believed that those younger than 75 years have the potential to become more self-sufficient with the right intervention. Therefore, the age limit was set to target those who could most benefit from this specific approach.

**Inclusion and exclusion criteria.**

**Inclusion criteria**

- Men and women whose native language is Arabic, Turkish, or Urdu with limited to moderate proficiency in Danish.
- Already in treatment at the outpatient type 2 diabetes clinic at Steno Diabetes Center Copenhagen, Denmark.
- Having suboptimal glycemic control (hemoglobin A_1c_ [HbA_1c_] ≥53 mmol/mol).
- Individual treatment goal for HbA_1c_ has not been achieved (in 2 consecutive measurements).

**Exclusion criteria**

- Being older than 75 years at inclusion.
- Residing part-time in Denmark.
- Having disabilities that inhibit physical attendance.
- Having severe mental disorders.
- Having an estimated glomerular filtration rate below 30 mL/min/1.73 m².
- General condition contraindicates continuing the study, judged by the investigator or medical expert.
- Withdrawal of the informed consent.
- Other reasons determined by the investigator.

### Recruitment, Screening, and Consent

We plan to recruit 96 Arabic-, Turkish-, or Urdu-speaking individuals with T2D from the outpatient T2D clinic at SDCC. During the latest visit to the usual course of treatment at SDCC, potential participants will receive a brief introduction to the person-centered and culturally sensitive course of treatment in the type 2 diabetes (ACCT2) study. If interested, potential participants will be met by a project worker who will provide an oral introduction and, if interested, play an audio recording containing study information in Urdu, Arabic, or Turkish, depending on the participant’s preference. All study participants will receive written study information in their native language. The information will be given in quiet surroundings at SDCC. The ability to bring one companion is allowed.

The project worker will have a written informed consent ready to sign and be available to address any questions. Participants may have at least 24 hours to consider participation. A participant folder containing the written study information and the written informed consent will be handed to the potential participant, and a project worker will follow up with a phone call within a week. If the participant wishes to take part in the study, the written informed consent must be signed and forwarded to the project worker. By January 16, 2025, a total of 88 out of 96 participants were recruited. The recruitment process will continue until March 2025.

To ensure accessibility, all written information is translated into Urdu, Arabic, or Turkish by international standards [30]. Study participants are informed that they can always withdraw their commitment to participate. The individual participant’s right not to know their data will be respected.

### Randomization

**Figure 1:**
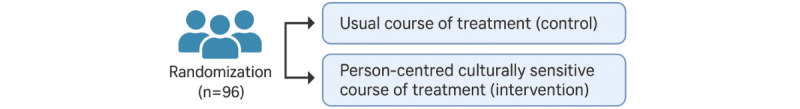
Randomization.

After obtaining written informed consent, participants will be randomized to either the intervention or control group in a 1:1 ratio using block randomization (Figure 1). Block randomization is used to secure an equal distribution of participants in the 2 groups if the study, for unexpected reasons, should be terminated before inclusion of all participants, and further to account for potential seasonal confounding.

Randomization will be performed by the investigator before the first visit using the randomization module in the software REDCap (Research Electronic Data Capture; Vanderbilt University). The randomization list is created by a researcher not involved in the trial, who will be the sole person to access it throughout the trial. Should this person be unable to continue this function, a new person not involved in the trial will be appointed to store and access the sequence. Participants are recruited continuously.

### Patient and Public Involvement

The intervention was developed in collaboration with the target group and HCPs from the outpatient T2D clinic at SDCC. Overall, the ACCT2 study consists of three phases: (1) needs assessment, (2) intervention development and element testing, and (3) RCT.

Phase 1 (needs assessment) included interviews and workshops with Arabic-, Turkish-, or Urdu-speaking individuals with T2D (n=18) and 2 workshops and individual interviews with HCPs (nurses, physicians, and dieticians) from the SDCC outpatient clinic (n=33). The findings from the needs assessment suggested adjustments in the standard course of treatment by focusing on (1) extended time in consultations, (2) use of technology for diabetes monitoring, (3) awareness of language barriers, (4) meeting the same HCP, (5) use of accurate communication and dialogue tools, and (6) a need for increased levels of health literacy. A culturally sensitive approach of HCPs was also considered vital to increase understanding and engagement in diabetes management among the target group while emphasizing a whole-person approach rooted in individual everyday life.

Based on the results from the needs assessment (unpublished), a 1-year intervention was designed to target the target group’s needs. Visits in the intervention design were tested on 3 individuals speaking Urdu, Arabic, and Turkish as their native languages to test if the flow, content, and overall design match their needs. Tools and methods included in the intervention are partly inspired by the research-based diabetes self-management education, Culturally Sensitive Tools and Methods (CUSTOM) for individuals with non-Western backgrounds [31,32]. The RCT design is described in this study (phase 3). The CONSORT-EHEALTH (Consolidated Standards of Reporting Trials of Electronic and Mobile Health Applications and Online Telehealth) reporting guidelines were applied (Multimedia Appendix 1) [33].

### RCT Study Design

**Figure 2:**
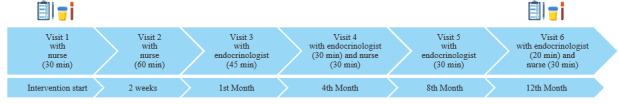
Visit flow.

The ACCT2 study is a 1-year pragmatic randomized controlled parallel group open-label effectiveness trial including 96 Arabic-, Turkish-, or Urdu-speaking individuals with T2D (Figure 2). The intervention consists of 6 visits to SDCC. All visits are based on specific values and competencies of HCPs, which are important in the organization, implementation, and completion of the intervention.

### Values of the Intervention

The intervention is based on a person-centered approach [34,35]. A person-centered approach is characterized as a collaborative process between the participant and the HCP, wherein the participant’s priorities, preferences, and needs are incorporated into decisions regarding their diabetes treatment. It focuses on understanding the patient’s capacity and pace for change [34,35], where cultural competence among HCPs is crucial. This includes adapting attitudes to individual needs and recognizing the implications of emotional, cultural, social, and psychological consequences [17]. Striking a balance, HCPs must acknowledge potential cultural differences without letting them dominate encounters. The intervention’s visits consider individual circumstances, resources, expectations, and prerequisites for changes. In addition, it aligns with the empowerment treatment philosophy, where well-informed participants actively engage in diabetes management. The HCP’s role is to support the participant in making decisions, achieving goals, and overcoming obstacles [36].

### Study Visits

The visits will be run by the same diabetes nurse and endocrinologist under the responsibility of the investigator. Visits with dieticians will be arranged if requested by participants (up to 60 min per visit). For logistical reasons, windows are allowed for the visits (±30 days). The intervention staff includes the same diabetes nurse and endocrinologist, each overseeing the 48 participants in the intervention group. The 48 control participants will continue their care with their usual HCPs. A detailed visit flow is presented in Figure 2.

#### Visit 1

The first visit, lasting 60 minutes, will involve meeting with a diabetes nurse. During this visit, the focus will be on introducing the participant to the course of treatment and to a continuous glucose monitor (CGM; FreeStyle Libre 2 sensor), which the participant will use for the following 2 weeks. This will enable the participant to gain insight into personal glucose levels. In addition, the nurse will provide information about T2D and its treatment and discuss everyday challenges and topics relevant to the participant.

#### Visit 2

The second visit, lasting 60 minutes, will involve the same diabetes nurse. The main aim is to talk about pattern identification obtained from the CGM sensor for the past 2 weeks to enhance knowledge of lifestyle factors contributing to stable or fluctuating glucose levels. The nurse will engage in a dialogue about the challenges participants face in everyday life. A new sensor will be applied to the participant for the upcoming 2-week period.

#### Visit 3

During the third visit, lasting 45 minutes, the participant will meet with an endocrinologist. Patterns derived from the CGM sensor will be discussed to enhance participants’ comprehension of unique glucose levels and determine if any changes or solutions discussed during the second visit have been implemented and proven effective in promoting more stable glucose levels. The endocrinologist will evaluate patterns of variation, for example, focus on hypoglycemia, and make necessary adjustments in pharmacological treatment. The treatment plan will be negotiated with the participant, providing information on the rationale for the change in treatment regimen and potential benefits and risks. The participant will also be guided to continue self-monitoring of blood glucose according to individual plans.

#### Visit 4

During the fourth visit (2x30 mins), the participant will meet with the same endocrinologist and diabetes nurse. The main purpose is to evaluate and follow up on the participant’s everyday life with diabetes, medication use, and individual needs. The aim is to support the participant’s positive experience with the treatment and lifestyle changes, address misperceptions, and reinforce the information provided. If necessary, the endocrinologist will adjust the medication regimen.

#### Visit 5

During the fifth visit, lasting 30 minutes, the participant will meet with the same endocrinologist. The main purpose is to evaluate and follow up on the participant’s medication usage and individual needs. The aim is again to support the participant’s positive experience with the treatment and lifestyle changes, address misperceptions, and reinforce the information provided. If necessary, the endocrinologist will adjust the medication regimen.

#### Visit 6

During the sixth visit (20+30 min), the participant will again meet the same endocrinologist and diabetes nurse. The primary aim is to provide a comprehensive overview of the treatment progress and patterns obtained from the CGM sensor for the past 2 weeks. The participant will be instructed to apply the sensor at home 2 weeks before the last visit. If the participant prefers, they can be booked for a short visit at SDCC with the diabetes nurse to apply the sensor. The last visit will serve as an opportunity to ask any questions, clarify doubts, or express any specific needs the participants may have.

Besides the 6 visits, the participant will receive links to 4 short videos about T2D developed by the Danish Diabetes Knowledge Center at SDCC. The videos are translated into Urdu, Arabic, or Turkish and include knowledge on (1) T2D, (2) complications associated with T2D, (3) exercise and diabetes, and (4) carbohydrates and diabetes (for details on content, refer to [37]). Participants in the intervention group may also have visits, for example with a psychologist and a podiatrist, as part of their usual course of treatment.

To minimize the risk of intervention contamination, HCPs in the study will not interact with participants in the control group. The control group does not have access to any materials, technology, or other components provided to the intervention group. This will ensure that the 2 groups remain distinct, preventing cross-contamination and preserving the integrity of the intervention’s evaluation.

### Control Group

**Figure 3:**
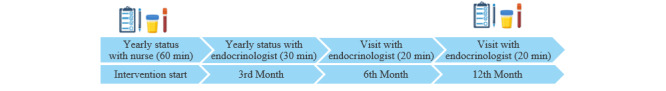
Standard course of treatment over 1 year.

Participants in the control group will follow the standard course of treatment, which includes regular visits with a physician, often an endocrinologist, and a nurse at the T2D outpatient clinic at SDCC 3-4 times per year (Figure 3). These visits can be in-person or computer-based, depending on the preferences of the participant and HCPs. The standard course of treatment also often includes visits with a podiatrist and eye screening, and it may also include visits with a dietician and psychologist.

### Outcomes

#### Primary Outcome

The primary outcome is changes in HbA_1c_ (mmol/mol) from baseline to the end of the 1-year intervention.

#### Secondary Outcomes

The secondary exploratory outcomes encompass a range of metabolic and behavioral factors that may be linked to the intervention. These involve changes from baseline to the end of the intervention (1 year), including plasma lipids, markers of kidney function, hormones involved in glucose metabolism, blood pressure, resting heart rate, CGM (time-in-range, time-above-range, and time-below-range), body weight, and use of diabetes medication (Table 1).

**Table 1:** Data collection.

Visit sequence number			Visit 1		Visit 2		Visit 3		Visit 4		Visit 5		Visit 6
**Clinical assessments**													
	HbA_1c_^a^	✓						✓		✓		✓	
	Plasma lipids	✓										✓	
	Blood pressure and resting heart rate	✓				✓		✓		✓		✓	
	Hormones involved in glucose metabolism	✓										✓	
	CGM^b^			✓		✓						✓	
	Markers of kidney function	✓										✓	
	Albumin-creatinine ratio	✓										✓	
	Body weight	✓										✓	
	Use of diabetes medication	✓										✓	
**Questionnaires**													
	Sociodemographic characteristics	✓											
	Diabetes distress (PAID-5^c^ scale) [38]	✓										✓	
	Well-being (WHO-5^d^) [39]	✓										✓	
	HLQ^e^ (3 items) [40]	✓										✓	
	Diabetes management (PRO^f^ scheme diabetes) [41]	✓										✓	
	MARS-5^g^ [42]	✓										✓	
	Acceptability, retention, and satisfaction [43]											✓	
**Interviews**													
	Individual interviews (subgroup of participants)							✓				✓	
	Interviews with HCPs^h^ delivering the intervention							✓				✓	

^a^HbA_1c_: hemoglobin A_1c_.

^b^CGM: continuous glucose monitoring.

^c^PAID-5: Problem Areas in Diabetes Scale–5.

^d^WHO-5: World Health Organization–Five Well-Being Index.

^e^HLQ: Health Literacy Questionnaire.

^f^PRO: patient-reported outcome.

^g^MARS: Medication Adherence Rating Scale.

^h^HCP: health care professional.

### Clinical Assessments

#### Blood Samples

Blood samples are, as part of the regular treatment protocol at the outpatient clinic in SDCC, routinely taken before visits. The participant must arrive at the SDCC laboratory at least 70 minutes before visit 1 and visit 6 for nonfasting blood sampling from the antecubital vein and urine analysis.

#### CGM Use

CGM is initiated using the authorized FreeStyle Libre 2 device. Participants will be provided with detailed information about the device during visit 1. It will be applied for 2 weeks. The monitoring will be repeated at visit 2 and again 2 weeks before visit 6. FreeStyle Libre 2 allows individuals to monitor glucose levels without routine fingerstick blood glucose testing. It is a small sensor worn on the skin that measures glucose levels in the interstitial fluid to display real-time glucose readings. With features such as trend arrows and built-in memory for data storage, FreeStyle Libre 2 supports diabetes management and provides insights into glucose patterns to inform decisions about medication, diet, and lifestyle.

#### Blood Pressure and Resting Heart Rate

Blood pressure and resting heart rate are measured using approved and calibrated equipment (Microlife BP A3 Plus) and taken with the participant after a minimum of 10 minutes of rest, without talking during the measurement. Measurements are repeated 3 times, separated by 2-minute breaks to prevent artificially elevated blood pressure resulting from an unfamiliar and potentially stressful environment. The mean value is calculated.

#### Height and Body Weight

Height is measured with the participant’s heels, buttocks, and upper back remaining in contact with the wall. Results are noted to the nearest 0.1 cm. Body weight is measured to the nearest 0.1 kg, with the participant wearing indoor clothes and no shoes.

#### Questionnaires

Participants will fill in a baseline questionnaire and a questionnaire at the end of the intervention (refer to Table 1). Questionnaires will be answered during the 70-minute waiting time between blood samples before visit 1 and visit 6. A project worker will be present to address technical questions. Questionnaires are translated into Urdu, Arabic, or Turkish following international standards [30]. The following measures will be assessed at baseline alone: marital status, cohabitation status, social network, educational level, and occupation. Participants’ acceptance of the intervention is only explored in the questionnaire at the end of the intervention.

#### Interviews

Interviews will be conducted by a qualitative researcher with a subgroup of participants in the intervention group after the last visit. Participants’ experiences during the intervention will be explored to examine experiences with the 1-year intervention, possible improvements, and important factors for successful implementation into the clinical setting. HCPs delivering the intervention will also be interviewed about acceptability and experiences with intervention delivery in the clinical context.

#### Measurement of Adherence

Adherence to the intervention is determined by calculating the number or percentage of visits during the intervention in which participants actively engage in all 6 scheduled visits. Per protocol is defined as ≥80% adherence.

### Statistical Considerations

#### Sample Size Calculation

A 5.0-mmol/mol change is the minimal difference for the primary outcome, HbA_1c_. This resulted in the total number of participants required to enter and complete this 2-arm, parallel-design trial of 88 participants. The sample size was estimated using a general linear model equivalent to a repeated measures mixed model with 2 time points (PROC GLMPOWER, SAS 9.4; SAS Institute). The estimation was based on the following conditions: an allocation ratio of 1:1, **α**=.05, **β**=.1, a mean HbA_1c_ level of 70 (SD 14) mmol/mol at each timepoint [44,45], and a correlation between repeated measures of 0.8 [46]. The total number of participants was multiplied by 1.1 to account for potential uncertainties in the conditions used in the power calculation and to account for potential dropouts, resulting in 96 participants.

#### Data Management

Data will be entered directly into electronic case report forms using REDCap, licensed by the Capital Region of Denmark, or saved electronically. All forms are filled out during (or immediately after) each visit. Errors and corrections are logged as provided by the REDCap interface. For CGM, source data will be registered in web-based software (LibreView, Abbott).

#### Statistical Analysis

An intention-to-treat analysis, encompassing all randomized participants, will be conducted upon completion of the last participant’s final visit. A per-protocol analysis will be carried out, including participants who demonstrate compliance throughout the intervention. Data will be presented using standard descriptive statistics, with means and SDs for normally distributed data and medians with IQRs for data not conforming to normal distribution. Changes from baseline and distinctions in delta values between groups will be assessed using linear mixed-effects models, considering the outcome as a function of group, time, and the interaction between group and time, and accounting for a participant-specific random intercept. The adequacy of assumptions regarding normality and homogeneity of variances will be verified through graphical methods, and data will be log-transformed for analysis and subsequently back-transformed for presentation. In cases where model assumptions are not met even after logarithmic transformation, nonparametric statistical tests will be used. Statistically significant findings will be determined with *P*<.05 (2-tailed). To assess the potential impact of missing data on the primary outcome, a sensitivity analysis will be conducted, involving multiple imputations with missing data at follow-up, which will be included in the imputation process with the control group. Results will be presented as estimated mean changes, accompanied by 95% CIs, degrees of freedom, and, if appropriate, the test value.

Covariables such as age, sex, diabetes duration, and so on, will be included in analyses to assess their potential influence on the outcomes. This approach will allow us to adjust for confounding factors and provide an accurate estimation of the intervention's impact. In addition, if the intervention proves effective, a health economic evaluation will be conducted to estimate the cost per person. This evaluation will consider direct costs, for example, staff time and technology, alongside potential cost savings from improved health outcomes, reduced complications, and fewer health care visits.

### Risk of Harm

All equipment used in the study meets the requirements for patient safety and has previously been used in research projects. Participants will have blood samples taken before visits (maximum 18 mL). The total amount of blood collected during the entire study period is at the same level as during the standard course of treatment and is considered safe.

Participants will not have any study-specific clinical measurements taken besides three 2-week periods of CGM measurement. During the sensor placement, participants may experience minor and brief discomfort. The sensor is generally well-tolerated, and only a few potential discomforts are associated with its use. Some participants may experience mild skin irritation or redness where the sensor is inserted. There may be a slight discomfort during the insertion process, although it is generally brief. In rare cases, an allergic reaction to the sensor adhesive or materials may occur, leading to more significant skin irritation. It is also worth noting that the sensor needs to be replaced twice, which involves removing the old sensor and applying a new one, which can cause minor discomfort. However, these discomforts are small and temporary. A rare complication is a superficial skin infection obtained in connection with penetration of the skin. The risk of superficial phlebitis is minimized by following hygiene standards, including double cleansing of the skin with an alcohol swab and using sterile materials. Superficial phlebitis is not dangerous. Medication will be reviewed and adjusted according to current clinical guidelines. Participants can contact the physician and diabetes nurse or use the hotline at SDCC about medical issues, for example, concerns related to medication use.

### Dissemination

Appointed researchers affiliated with SDCC will be granted access to data. The principal investigator will facilitate direct access to source data and documents for regulatory inspection. Since we anticipate no adverse effects associated with the intervention, recording of harm or side effects will be conducted. Positive and negative study results and inconclusive findings will be presented at conferences and published in international peer-reviewed journals. All coauthors are expected to adhere to the guidelines of the International Committee of Medical Journal Editors, and no professional writers will be involved in the writing process.

### Ethics Approval

The study has been approved by the Ethics Committee of the Capital Region of Denmark (H-23042245) and the Research Legal Affairs of the Capital Region of Denmark (P-2023-14503). The study will be conducted following the Declaration of Helsinki and will comply with the Danish Data Protection Agency and the General Data Protection Regulation. Adequate blinding of all personal data during data processing and publication will be ensured. The participants are covered by the Patient Compensation Association according to the Danish Act on the Right to Complain and Receive Compensation within the Health Service.

## Results

Study funded in June 2023. By March 2025, all participants were successfully enrolled in the study. Data collection will proceed until December 2025, allowing for comprehensive data gathering. Once data are collected, the research team will begin the analysis process, with the first results anticipated to be available by March 2026.

## Discussion

### Comparison With Prior Results

Despite the existing gaps in the literature, it is well-established that the target group faces an elevated risk of T2D [1-3], often attributed to factors such as health behavior, limited health care access, social determinants of health, low health literacy, cultural norms, and language barriers [4,9-16]. Our study aims to build on this understanding by using several robust methodologies. The use of a scientifically rigorous design, power calculation for sample size estimation, and a replicable intervention are among the strategies used. A control group allows for a meaningful comparison to “usual care,” enhancing the reliability of our findings. In addition, administering the intervention through the same HCPs ensures continuity in care and familiarity for participants, which may reduce dropout rates. A distinctive feature of our approach is the systematic involvement of the target group and HCPs in the intervention’s design. By incorporating their insights into their needs, barriers, and potential solutions, we aim to create an intervention that better addresses their challenges than previous research. This tailored approach could lead to improved diabetes management, enhanced well-being, and reduced obstacles that typically hinder care within this high-risk population.

### Strengths and Limitations

The interdisciplinary nature of our study, coupled with feasibility assessments, offers valuable insights into the experiences of both participants and HCPs, shedding light on potential barriers and effective strategies for implementing the intervention in outpatient clinics. However, it is important to acknowledge certain limitations. The 1-year duration of the trial restricts the exploration of long-term effects. In addition, the broad inclusion criterion, while facilitating a more representative study population, may introduce heterogeneity in some secondary outcomes. Also, the inherent challenge of blinding participants poses a potential for performance bias, which we cannot eliminate from the study design.

Moreover, we compare the intervention group to the usual care group, which, by definition, represents the standard treatment available. The intervention group, however, received additional resources and structured support, which may have led to greater improvements. This is a common approach in intervention studies, where the objective is to assess the added value of the intervention. While the observed benefits in the intervention group may be partly attributed to the enhanced support provided, it is essential to evaluate whether these improvements can be directly linked to the intervention itself. Therefore, intervention scalability, if it proves effective, includes further evaluations. Key considerations for scalability include assessing cost-effectiveness, resource allocation, and how the intervention can be integrated into routine clinical practice. We also plan a comprehensive realist evaluation, which will provide additional insights into the implementation and feasibility of the intervention. This evaluation will explore the perspectives of participants, their relatives, and HCPs with a focus on understanding what works for whom, why, and under what circumstances. By examining the mechanisms driving the intervention’s success or failure, we aim to identify the conditions and contexts necessary for effective implementation.

### Conclusions

The intervention design, tailored to address the needs of both the target group and HCPs, is poised to maximize its potential for acceptability within the target group, particularly should the outcomes prove positive. This study is expected to provide knowledge translatable to clinical settings, with the anticipated clinical impact extending to the future development and implementation of interventions adapted to preferences, needs, and satisfaction.

## Appendix

Multimedia Appendix 1 CONSORT-eHEALTH checklist (V 1.6.1).

## Abbreviations

ACCT2: a person-centered and culturally sensitive course of treatment in type 2 diabetes
CGM: continuous glucose monitor
CONSORT-EHEALTH: Consolidated Standards of Reporting Trials of Electronic and Mobile Health Applications and Online Telehealth
CUSTOM: Culturally Sensitive Tools and Methods
HbA_1c_: hemoglobin A_1c_
HCP: health care professional
RCT: randomized controlled trial
REDCap: Research Electronic Data Capture
SDCC: Steno Diabetes Center Copenhagen
T2D: type 2 diabetes

## Data Availability

Data sharing does not apply to this article, as no datasets were generated or analyzed during this study.
